# Predicting unipolar and bipolar depression using inflammatory markers, neuroimaging and neuropsychological data: a machine learning study

**DOI:** 10.1192/j.eurpsy.2023.1292

**Published:** 2023-07-19

**Authors:** L. Raffaelli, F. Colombo, F. Calesella, L. Fortaner-Uya, I. Bollettini, C. Lorenzi, E. Maggioni, E. Tassi, S. Poletti, R. Zanardi, F. Attanasio, F. Benedetti, B. Vai

**Affiliations:** 1 Division of Neuroscience, in vivo structural and molecular neuroimaging unit, IRCCS San Raffaele Scientific Institute, Milan, Italy; 2 Department of Eletronics Information and Bioengineering, Politecnico di Milano; 3 Division of Neuroscience, Neuropsycopharmacology Unit, IRCCS San Raffaele Scientific Institute, Milan, Italy

## Abstract

**Introduction:**

About 60% of bipolar disorder (BD) cases are initially misdiagnosed as major depressive disorder (MDD), preventing BD patients from receiving appropriate treatment. An urgency exists to identify reliable biomarkers for improving differential diagnosis (DD). Machine learning methods may help translate current knowledge on biomarkers of mood disorders into clinical practice by providing individual-level classification. No study so far has combined biological data with clinical data to provide a multifactorial predictive model for DD.

**Objectives:**

Define a predictive algorithm for BD and MDD by integrating structural neuroimaging and inflammatory data with neuropsychological measures (NM). Two different algorithms were compared: multiple kernel learning (MKL) and elastic net regularized logistic regression (EN).

**Methods:**

In a sample of 141 subjects (70 MDD; 71 BD), two different models were implemented for each algorithm: 1) structural neuroimaging measures only (i.e. voxel-based morphometry (VBM), white matter fractional anisotropy (FA), and mean diffusivity (MD)); 2) VBM, FA, and MD combined with NM. In a subsample of 71 subjects (36 BD; 38 MDD), two similar models were implemented: 1) VBM, FA, and, MD combined with only NM; 2) VBM, FA, and MD combined with NM and peripheral inflammatory markers. Finally, the best model was selected for comparison with healthy controls (HC).

**Results:**

Overall, the EN model based on all the modalities achieved the highest accuracy (AUC = 90.2%), outperforming MKL (AUC=85%). EN correctly classified BD and MDD with a diagnostic accuracy of 78.3%, sensitivity of 75%, and specificity of 81.6%. The most significant predictors of BD (variable inclusion probability (VIP) > 80%) were the parahippocampal cingulate, interleukin 9, chemokine CCL5, posterior thalamic radiation, and internal capsule, whereas MDD was best predicted by chemokine CCL23, the anterior cerebellum, and the sagittal stratum. In contrast, NM did not help to differentiate between MDD and BD. However, they help to distinguish patients from HC. Psychomotor coordination and speed of information processing discriminated between MDD and HC (VIP>90%), whereas fluency, working memory, and executive functions differentiated between BD and HC (VIP>80%).

**Conclusions:**

In summary, BD was predicted by a strong proinflammatory profile, whereas MDD was identified by structural neuroimaging data. A multimodal approach offers additional instruments to improve personalized diagnosis in clinical practice and enhance the ability to make DD.

**Disclosure of Interest:**

None Declared

